# Benign acute childhood myositis: a retrospective cohort study from a large tertiary care children's hospital

**DOI:** 10.3389/fped.2025.1653651

**Published:** 2025-09-09

**Authors:** Nawaf A. Alghamdi, Abdulaziz A. Ajeebi, Abdulrahman K. Alajlan, Abdullah Y. Aldaffaa, Winnie Philip, Tahir K. Hameed

**Affiliations:** ^1^ Department of Pediatrics, King Abdullah Specialized Children’s Hospital, King Abdulaziz Medical City—Riyadh, Ministry of National Guard Health Affairs, Riyadh, Saudi Arabia; ^2^ King Abdullah International Medical Research Center, Ministry of National Guard Health Affairs, Riyadh, Saudi Arabia; ^3^ College of Applied Medical Sciences, King Saud bin Abdulaziz University for Health Sciences, Riyadh, Saudi Arabia; ^4^ College of Medicine, King Saud bin Abdulaziz University for Health Sciences, Riyadh, Saudi Arabia

**Keywords:** benign acute childhood myositis, viral myositis, influenza, child, rhabdomyolysis

## Abstract

**Introduction:**

Benign acute childhood myositis (BACM) is a common self-limiting condition. While studies in other regions have described the epidemiology and outcomes of BACM, there is paucity of data in the Middle East Region. This study aims to describe the epidemiology, clinical data, and outcomes of BACM in a large cohort of patients.

**Methods:**

This was a retrospective cohort study of children diagnosed with BACM at a tertiary care children's hospital between January 2016 and December 2022. The study included children under 14 years with acute onset of muscle pain with elevated CK levels. Clinical, laboratory and outcome data were extracted from the medical records.

**Results:**

A total of 392 children were diagnosed with BACM, with a male predominance (78.6%) and a median age of 6 years. Median CK level at presentation was 1,750 U/L and an Influenza virus was found in 92.8% of those who had a virus detected. Rhabdomyolysis was diagnosed in 4 (1%) patients and no cases of renal failure were reported. CK levels >5,000 U/L on presentation increased the risk for hospitalization while ibuprofen use in the ED decreased the risk of hospitalization. CK levels normalized at a median time of 7 days and recurrences with new episodes of BACM occurred in almost 10% of children.

**Conclusions:**

Our study confirms that BACM is a benign condition with a very low rate of complications. Further studies are needed to evaluate factors associated with hospitalization and when to screen for genetic/metabolic causes of elevated CK levels.

## Introduction

Benign acute childhood myositis (BACM), also known as viral myositis, is a common condition seen in the emergency department (ED) and a frequent cause of hospital admission in pediatrics. It is characterized by localized muscular pain (especially in the calves) that typically follows a viral upper respiratory tract infection (1). The most common virus implicated in BACM is influenza virus (1–4). The association of viral illness with muscular pain was first described in 1957 by Lundberg, when he found that affected children often came from the same family or same school class (5).

BACM is usually diagnosed based on findings of bilateral calf muscle pain and tenderness and sometimes refusal to walk that was preceded a few days earlier with typical flu-like symptoms. An increase in the level of the enzyme creatinine kinase (CK), sometimes in the thousands, is often found (2). Inflammatory markers are usually normal and there may be mild leukopenia and thrombocytopenia secondary to the causative viral agent (6, 7). Renal function is usually preserved in this condition (3, 6). Although BACM is almost always a self-limiting condition with recovery in 3–5 days (1), it should be differentiated from more serious conditions like rhabdomyolysis. This condition is characterized by leakage of intracellular contents such as electrolytes, myoglobin, and creatine kinase into the blood circulation. CK elevations tend to range from 5 to more than 50 times the upper limit of normal, and the condition can lead to renal failure (7). While one study from 2 tertiary care centers found no cases of rhabdomyolysis in children with BACM (7), a recent study found a rate of almost 7% (8). A few studies have looked at the recurrence rate of BACM and found it to be 3%–10% (1, 9, 10). BACM is considered a self-limiting condition, and the recommended management is symptomatic treatment with analgesia as needed and good hydration (2).

While several studies from Europe and Asia have described the clinical and laboratory characteristics of BACM, there are few published studies from the Middle East Region. One study from Israel of 54 patients found that the mean CK level on presentation was 1,872 U/L and no patients developed renal failure (7). A recent study from Saudi Arabia on 297 children found 3 patients (1.1%) had elevated serum creatinine levels. Of note though, almost half the patients in this study had CK levels of less than 240 U/L (11). Despite BACM being a common condition, there are no clear guidelines for investigations in patients (12). The aim of our study was to describe the epidemiology, clinical characteristics, investigations and outcomes of BACM in a large cohort of children presenting to the ED. We also sought to determine factors associated with hospitalization and when further investigations are warranted in patients with BACM.

## Methods

### Study design

This is retrospective cohort study of children under 14 years of age who presented to the ED at King Abdullah Specialized Children's Hospital—Riyadh, Ministry of National Guard Health Affairs, and were diagnosed with Benign Acute Childhood Myositis (BACM). The study period was 7 years (2016–2022). BACM was defined as acute onset of muscle pain in a child with elevated serum CK level of 200 U/L or more. CK level ≥200 U/L was used as this was the upper limit of normal reported in our laboratory and consistent with the cut-off for abnormal CK levels reported in other recent studies (1, 3). Inclusion criteria were patients under 14 years of age presenting to the ED and diagnosed with BACM. Patients were excluded if they had a diagnosis of another condition associated with elevated CK level (e.g., Juvenile dermatomyositis, muscular dystrophies, etc.). Patient charts of those meeting inclusion criteria were reviewed in detail including demographic, clinical, and laboratory data. Laboratory data analyzed included CK levels, renal function tests, urinalysis/urine dipstick, and respiratory multiplex PCR tests. CK level on presentation was the CK level ordered in the ED, while peak CK level was the highest CK level in admitted patients. Respiratory multiplex PCR panel tests from nasopharyngeal swab/aspirate specimens were done at the discretion of the admitting physician. Viruses tested in this panel include common viruses associated with viral myositis such as influenza viruses, parainfluenza viruses, adenovirus, and SARS-CoV-2. In addition, all charts were reviewed to determine outcomes including recurrences.

### Statistical analysis

Data collection and analysis were conducted using Microsoft Excel and SPSS version 21. Categorical data, including patient demographics and clinical presentation, were reported using frequencies and percentages. Continuous data were expressed with means and standard deviations used for normally distributed data (as confirmed by the Shapiro–Wilk normality test) and medians and interquartile ranges (IQR) applied to skewed data. Logistic regression analysis was used to assess the risk factors associated with hospitalization, including age, sex, CK level on presentation, and use of ibuprofen in the ED. CK level of >5,000 U/L was used as a cutoff as this level may be an indication for admission (13).

## Results

During the study period, 392 children were diagnosed with BACM, 308 (78.6%) of which were male. The median age was 6 years, with an interquartile range (IQR) of 4–7 years. Three hundred and eighteen (81.1%) patients had a history of fever and the median duration of fever preceding onset of BACM symptoms was 3 (IQR 2, 4) days. The vast majority of patients (365; 93.1%) presented with calf muscle pain while 247 (63%) presented with a gait abnormality (the most common being inability to walk). The median CK level at presentation and the peak CK levels were 1,750 U/L (IQR 762.25, 3,405.75) and 1,871 U/L (IQR 796.75, 3,967.75), respectively. Sixty patients (15.3%) had CK levels of >5,000 U/L on presentation. The median creatinine level was 44 umol/L (IQR 42, 48) and median blood urea nitrogen was 3.4 mmol/L (IQR 2.7, 4). Urine dipstick was done in 215 (54.8%) patients, with erythrocytes positive in 33 (15.3%) (Table 1).

**Table 1:** Demographic, clinical, and laboratory characteristics of patients with benign acute childhood myositis.

Variable	Summary Statistics
Sex, *n*(%)	
Male	308 (78.6)
Age in years, median (IQR)	6 (4, 7)
Symptoms and Signs, *n* (%)^a^	
Preceding fever	318 (81.1)
Muscle pain	373 (95.2)
Bilateral calf pain	365 (93.1)
Gait abnormality	247 (63)
Inability to walk	206 (52.6)
Laboratory Findings	
Median CK at presentation, IQR (*n* = 392)	1,750 U/L (762.25, 3,405.75)
Median CK at peak, IQR (*n* = 392)	1,871 U/L (796.75, 3,967.75)
Median creatinine, IQR (*n* = 384)	44 umol/L (42, 48)
Median blood urea nitrogen, IQR (*n* = 392)	3.4 mmol/L (2.7, 4)
Urine dipstick (*n* = 215)	
Erythrocytes positive	33 (15.3)

^a^ The subjects have more than one symptom and sign.

CK, creatinine kinase.

**Figure 1:**
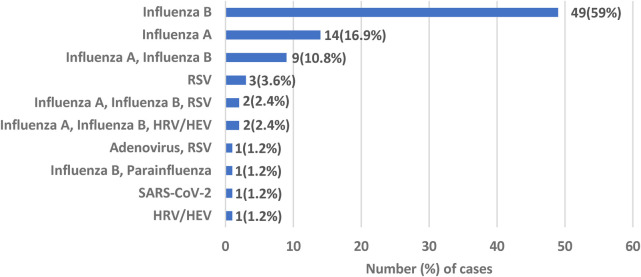
Viral etiology of benign acute childhood myositis. RSV, respiratory syncytial virus; HRV/HEV, human rhinovirus/human enterovirus.

In terms of etiology of BACM, 151 (38.5%) patients had respiratory viral testing. Viral testing was positive in 83 (55%) patients. Amongst the virus positive patients, 77 (92.8%) had at least one influenza virus detected. A total of 63 (75.9%) patients had Influenza B detected; 49 patients with this virus alone and 14 with a co-infection with another virus. Influenza A was detected in 27 (32.5%) patients; 14 with Influenza A alone and 13 as a co-infection with another virus. There were 3 patients (3.6%) with BACM due to RSV virus alone, 1 patient (1.2%) with human rhino/enterovirus and 1 patient (1.2%) with SARS-CoV-2 (Figure 1).

Of the 392 patients with BACM, 143 (36.5%) were admitted to the hospital. The median length of stay (LOS) for admitted patients was 2 days (IQR 1,2) and there was no correlation between CK levels and LOS (*p* = 0.171). Among the entire study population, 4 (1%) were diagnosed with rhabdomyolysis; CK levels on presentation ranged from 26,397 U/L to >42,670 U/L. One patient (0.3%) amongst the entire cohort of patients had a mildly elevated creatinine level on presentation. CK levels were retested in 77 (19.6%) patients. The median time to normalization of CK levels was 7 days (IQR 6, 10). Thirty-six patients (9.2%) had new episodes of BACM later in childhood (Table 2).

**Table 2:** Outcomes of patients with benign acute childhood myositis.

Variable	BACM Patients (*N* = 392), %
Admitted	143 (36.5)
Readmission following discharge	1 (0.7)
LOS (days), median (IQR)	2 (1,2)
Rhabdomyolysis	4 (1)
Elevated creatinine	1 (0.26)
CK level tested as outpatient	77 (19.6)
Recurrence	36 (9.2)

BACM, benign acute childhood myositis; LOS, length of stay.

Amongst those with CK levels exceeding 5,000 U/L at presentation, 42 (70%) were hospitalized. The likelihood of hospitalization was higher in males [odds ratio (OR) of 1.962 [95% CI 1.072, 3.590)] and in children with CK level on presentation >5,000 U/L [OR 4.955 (95% CI 2.458, 9.986)]. Two hundred and ninety-eight patients (76%) received ibuprofen in the ED. Of those who received ibuprofen in the ED, the likelihood of admission was lower; however it did not reach statistical significance [OR 0.590 (95% CI 0.328, 1.060)] (Table 3).

**Table 3:** Factors associated with hospitalization in patients with benign acute childhood myositis.

Variable	Hospitalization (*N* = 143)		
	Odds ratio (OR)	95% CI	*P* value
Age (≤5 years)	1.514	0.908, 2.524	0.112
Sex (male)	1.962	1.072, 3.590	0.029
CK level on presentation (>5,000 U/L)	4.955	2.458, 9.986	0.001
Ibuprofen used in the ED	0.590	0.328, 1.060	0.077

## Discussion

In this study, we described the epidemiology, clinical characteristics, and outcomes of children with benign acute childhood myositis (BACM) presenting to the ED in a large tertiary care children's hospital. Our main findings were that BACM is more common in boys aged 4–8, patients have a typical presentation, and influenza B is the most common causative virus. Almost all cases follow a benign course and the rate of complications was exceedingly rare. The optimal time to test CK levels as an outpatient was after 1 week.

In our study, we had a total of 392 patients with BACM, which translates to approximately 55 patients per year. Given that our ED has approximately 100,000 visits per year, the annual incidence of BACM in the pediatric ED is roughly 55 per 100,000. The exact incidence of BACM remains uncertain (1), and many children may not seek medical attention or are seen outside the ED (e.g., outpatient clinics). Boys accounted for the majority of the patients with BACM, consistent with the initial description of this condition by Lundberg (5) and in recently published studies (1, 3, 8). The exact reason of predominance of boys being affected more than girls is not known, but it has been postulated that it may be due to greater levels of activity in boys or genetic predisposition (14). Almost all patients presented with muscle pain (majority bilateral calf pain), more than three-quarters had preceding fever, and just over half had inability to walk. Our findings are consistent with classic characteristics of BACM presenting as sudden lower limb pain after an initial febrile period (3). We found the median time for development of BACM symptoms after onset of fever to be 3 days, consistent with a large study of influenza-associated BACM in Germany (15).

The median CK level on presentation was 1,750 U/L, consistent with findings in other recent studies (1, 3, 16). We observed that in those patients admitted, the CK level increased after admission with the peak CK level higher than the CK level on admission. This is expected as after a muscle injury, CK level peaks at approximately 2–5 days, and returns to normal in most patients about 1 week later (17). Influenza viruses were the most common etiology in BACM; amongst patients who had a viral etiology detected, over 90% were due to an Influenza virus, with Influenza B virus predominating. In a recent review of studies on BACM, Influenza B was the most common virus detected in BACM patients (2).

Amongst our cohort of patients, just over one-third of patients were hospitalized. The median LOS at 2 days was slightly shorter than length of stay reported in a recent review (2). Our study found that the rate of complications from BACM is very low; re-admissions are exceedingly rare, there were no cases of renal failure, and only 4 patients were diagnosed with rhabdomyolysis. Amongst the patients with rhabdomyolysis, one child was diagnosed with autosomal recessive myoglobinuria, acute recurrent. This child had a family history of a sibling with a similar presentation. Our finding of a low rate of rhabdomyolysis is consistent with most recently published studies (1, 3, 7), though one study reported that rhabdomyolysis developed in almost 7% of patients with BACM (8). Of note, there is no universally accepted definition of rhabdomyolysis (7), which makes comparisons of the rate of this complication difficult. While we found no cases of renal failure similar to other studies on BACM (3, 10, 15, 18, 19), one single-center of 78 patients with viral-induced rhabdomyolysis found the rate of acute kidney injury (AKI) close to 40% (20). This is in contrast to a multi-center study done in the United States on pediatric rhabdomyolysis hospitalizations reporting renal morbidity to be 8.5% (21). Given our large cohort of patients with many having very high CK levels, and only one patient having mildly elevated creatinine, we believe the risk of renal morbidity to be low in patients with BACM. Approximately one-fifth of patients had repeat CK levels as outpatients. The median time to normalization of CK level was 7 days, similar to what has been reported in the literature (3, 15). In our study we found that recurrence of new episodes of BACM to be relatively common, consistent with other studies (1, 10).

We explored factors affecting hospitalization and found that male sex and very high CK levels (>5,000 U/L) on presentation were associated with higher risk of hospitalization. BACM is known to be more common in males (2), and recent study showed a higher rate of hospitalization amongst males (1). We postulate that very high CK levels on presentation may influence the treating physician to hospitalize children more based on perceived higher risks of complications. Though not statistically significant, we found that ibuprofen may have a protective effect in decreasing the risk of hospitalization. Given that patients often present with severe calf pain and inability to walk, the analgesic effect of this medication may facilitate discharge from the ED. Further study is needed to evaluate any protective effect of ibuprofen and ensuring there is no development of acute kidney injury. Also, a recent study on 10 patients with myositis in the setting of COVID-19 found that children with a higher urea/creatinine ratio had longer times for CK levels to normalize (22). This correlation and whether the urea/creatinine ratio predicts risk of hospitalization or morbidity needs to be explored further especially in influenza-related myositis.

The findings from our study have many practical implications for the management of this common and self-limiting condition. We agree that basic blood tests including CK and urine dipstick to look for blood (which may indicate myoglobinuria) are usually all that is needed in most patients (7, 23). Next, ibuprofen use in the ED setting for this condition may decrease the rate of hospitalization. In addition, as renal failure is exceedingly rare in BACM, in those patients that are admitted, the common practice of “overhydration” may not be necessary, even in the setting of very high or rising CK levels. From our center's experience, many children after admission are clinically improving despite rising CK levels. Furthermore, we found that the optimal time to repeat CK level after discharge was after 1 week, with most patients having normal CK levels at this time period. Although indications to screen for inherited metabolic or muscular diseases in cases of BACM are not clear, we suggest to screen in cases of rhabdomyolysis. Up to 10% of cases of rhabdomyolysis may be due to underlying metabolic disorders (24). Also, as suggested by other authors, it may be reasonable if CK level does not fall or rises again after 2 weeks to proceed with screening (3).

Our study has many strengths. To the best of our knowledge, we present the largest cohort of patients with BACM. We were strict with our definition of BACM with all patients having elevated CK levels and excluding those patients with known conditions that can cause elevated CK levels (such as myopathies). In addition, we confirmed that BACM is a benign condition with a full recovery expected in patients and an exceedingly small risk of complications. Furthermore, our study described outcomes of patients and detailed the risk of recurrences and optimal time for repeating CK levels as an outpatient. The study does have limitations. First, though we present a large cohort of patients with BACM, this is a single center study. We also could not give a more objective assessment of acute kidney injury as we do not have previous serum creatinine level or details of the urine output in patients. Furthermore, given that only 83 patients of the entire cohort tested positive for viruses, and the number of Influenza A cases alone was low, we are unable to compare laboratory results, hospitalization rates, and outcomes between influenza viruses. Another limitation of the study is that we did not study other factors in detail regarding risk and outcomes of BACM including influenza vaccination status and the early use of Oseltamivir. Moreover, as the management of patients was not the focus of our study, we cannot give a detailed analysis of how many patients received “overhydration” and its effect if any on duration of normalization of CK levels.

## Conclusion

In this large cohort of patients with BACM, we confirmed that the condition is more common in boys, influenza B is the most common viral etiology, and patients had favorable outcomes. Despite significantly high CK levels in many patients, BACM remains a benign, self-limiting condition. Larger multi-center studies are needed to determine the safety and efficacy of ibuprofen in the ED setting to decrease hospitalization rates, factors predictive of complications, and the effect of influenza vaccine and anti-viral treatment in influenza-associated viral myositis.

## Data Availability

The raw data supporting the conclusions of this article will be made available by the authors, without undue reservation.
